# Supplementary material for: SARS-CoV-2-Specific Immune Cytokine Profiles to mRNA, Viral Vector and Protein-Based Vaccines in Patients with Multiple Sclerosis: Beyond Interferon Gamma
Source: Vaccines (Basel). 2024 Jun 19;12(6):684. doi: 10.3390/vaccines12060684 (PMC11209537; doi:10.3390/vaccines12060684)
Supplement: Supplementary file 1 [file vaccines-12-00684-s001.zip › vaccines-3032936-supplementary.pdf]

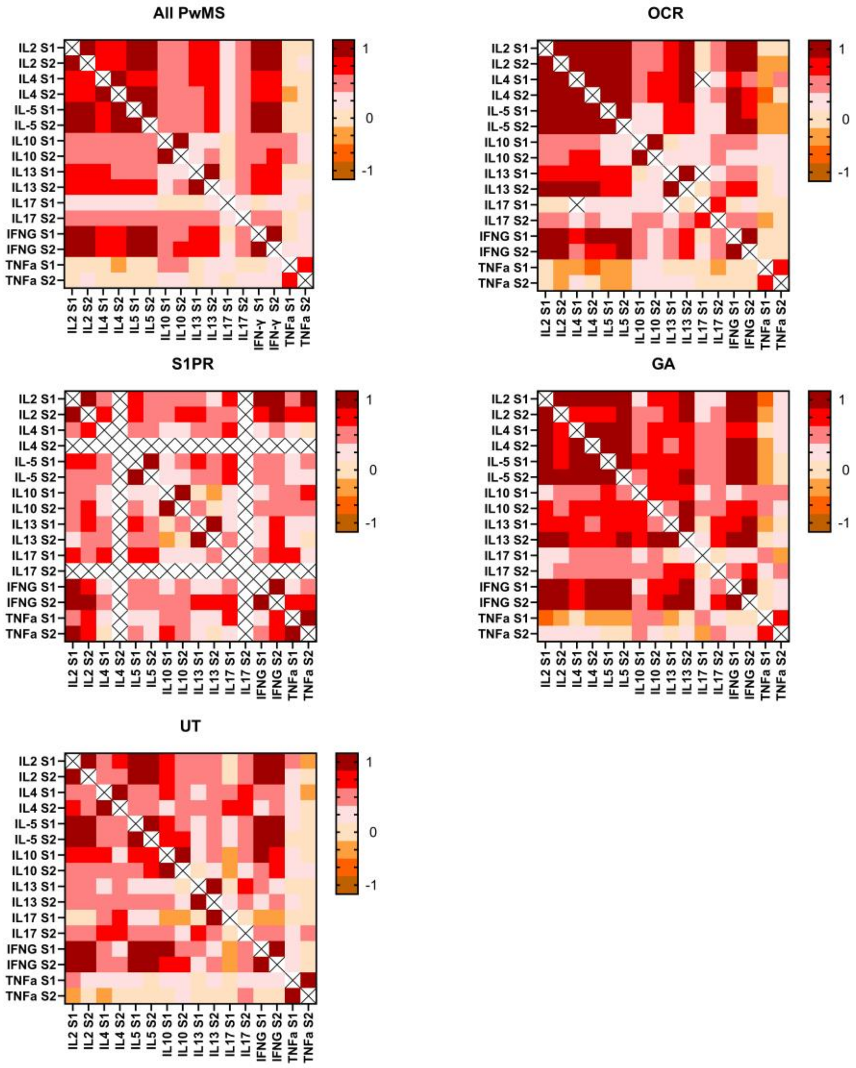

Figure S1. Correlation of cytokine profiles after stimulation with SARS-CoV-2 Ag1 and Ag2 after vaccination with two doses of mRNA or VVV. Different treatment modalities are depicted. Spearman's Rho Coefficient (ϱ) on a color gradient from -1 to +1 is presented. GA= glatiramer acetate; OCR= Ocrelizumab; S1PR= Sphingosine 1 Phosphate Receptor; UT= untreated.
